# ﻿*Orthotrichumcamanchacanum*, a remarkable new moss species from Chile (Bryopsida, Orthotrichaceae)

**DOI:** 10.3897/phytokeys.242.120717

**Published:** 2024-05-20

**Authors:** Vítězslav Plášek, Jakub Sawicki, Felipe Osorio, Monika Szczecińska, Hana Režnarová

**Affiliations:** 1 Department of Biology and Ecology, University of Ostrava, Ostrava, Czech Republic; 2 Institute of Biology, University of Opole, Opole, Poland; 3 Department of Botany and Nature Protection, University of Warmia and Mazury in Olsztyn, Olsztyn, Poland; 4 Universidad Austral de Chile, Instituto de Conservación, Biodiversidad y Territorio, Facultad de Ciencias Forestales y Recursos Naturales, Valdivia, Chile; 5 Museo de la Exploración Rudolph Amandus Philippi, Isla Teja Campus de Los Museos, Valdivia, Chile; 6 Laboratorio de Biodiversidad y Ecología del Dosel, Universidad Austral de Chile, Instituto de Conservación, Biodiversidad y Territorio, Valdivia, Chile

**Keywords:** Bryophytes, new taxa, Orthotrichaceae, South America, taxonomy

## Abstract

*Orthotrichumcamanchacanum* is presented as a newly described species from Chile. The species is primarily distinguished by its emergent capsule with cryptoporous stomata, a double peristome, linear-lanceolate stem leaves with a long hyaline aristae in apex, conspicuously differentiated perichaetial leaves, and a densely hairy vaginula. The species was discovered in the mountain massif of the Andes in the Coquimbo region, notable for its unique climatic conditions. Molecular data and a brief discussion comparing the newly described species with the most closely related taxa are also provided.

## ﻿Introduction

*Orthotrichum* Hedw. is a cosmopolitan moss genus, mainly distributed in temperate regions of both northern and southern hemispheres. Similarly, as is the case with most of such moss genera, *Orthotrichum* has been recognized as a heterogeneous taxon (Sawicki et al. 2017). Extensive taxonomic and molecular investigations have confirmed its polyphyly, resulting in the separation of three distinct segregates from the genus: *Nyholmiella* Holmen & E. Warncke; *Pulvigera* Plášek, Sawicki & Ochyra and *Lewinskya* F. Lara, Garilleti & Goffinet (Sawicki et al. 2009, 2010, 2017; Plášek et al. 2015; Lara et al. 2016). In South America, the genus *Orthotrichum* s. str. displays a widespread presence, primarily inhabiting the forests or bushes of temperate regions. These mosses predominantly thrive as epiphytes on trees and shrubs, occasionally as epilithic species on boulders and rocks (Lewinsky 1984).

*Orthotrichum* in Chile was comprehensively treated by Lewinsky (1984). Subsequent taxonomic findings and new distribution data were further documented by Lewinsky and Deguchi (1989), Müller (2002, 2009), Buck (2005), Ireland et al. (2006, 2017), Goffinet et al. (2007), Medina et al. (2013), Larraín (2016), Larraín and Bahamonde (2017), Drapela and Larraín (2020), Lara et al. (2021), and Ellis et al. (2023). According to these studies, *Orthotrichum* s. str. is there represented by eight species and two varieties (see also Table 1): *Orthotrichumanomalum* Hedw., *O.assimile* Müll. Hal., O.cupulatumvar.austroamericanum Lewinsky, O.diaphanumvar.podocarpi (Müll. Hal.) Lewinsky, *O.freyanum* Goffinet, W.R. Buck & M.A. Wall, *O.gigantosporum* Lewinsky, *O.inclinatum* Müll. Hal., *O.perexiguum* Dusén ex Lewinsky, *O.tristriatum* Lewinsky, and *O.truncatum* Lewinsky & Deguchi. This paper describes a new species from Chile, bringing the total number of *Orthotrichum* taxa in the country to eleven.

**Table 1.** Occurrence of *Orthotrichum* taxa in Chile based on literature data summarizing taxonomic and floristic research.

**Species / literature sources**	**Lewinsky (1984)**	**Lewinsky and Deguchi (1989)**	**Müller (2002)**	**Buck (2005)**	**Goffinet et al. (2007)**	**Müller (2009)**	**Buck and Goffinet (2010)**	**Medina et al. (2013)**	**Ireland et al. (2017)**	**Larraín (2016)**	**Larraín and Bahamonde (2017)**	**Drapela and Larraín (2020)**	**Lara et al. (2021)**	**Ellis et al. (2023)**
*Orthotrichumanomalum*				♢		♢					♢			
*O.assimile*	♢					♢			♢	♢	♢			
O.cupulatumvar.austroamericanum	♢					♢						♢		♢
O.diaphanumvar.podocarpi			♢			♢								
*O.freyanum*					♢	♢			♢					
*O.gigantosporum*		♢				♢							♢	
*O.inclinatum*	♢					♢	♢							
*O.perexiguum*	♢					♢			♢					
*O.tristriatum*	♢					♢		♢	♢					
*O.truncatum*		♢				♢			♢					

Previously, *Orthotrichumaequatoreum* was also reported from the territory of Chile (Ireland et al. 2006). However, a subsequent review of the material revealed that this was a misidentification (Ireland et al. 2017).

## ﻿Material and methods

### ﻿Plant material

During a bryofloristic survey in the Andes Mountains in 2021, a remarkable epiphytic moss from the genus *Orthotrichum* was collected. Specimens were carefully gathered, air-dried, and sent to the University of Ostrava for identification and inclusion in the herbarium collections (herbarium OSTR). The material proved to be the first record of this taxon for Chile and, upon closer examination, also to represent a species new to science. Plants were subsequently documented using an Olympus SZ61 trinocular microscope for macrophotographs and Olympus BX53 and IPOS-810 microscopes for microphotographs. Detailed SEM photo-documentation of peristome structures and spores was carried out using a Jeol SEM microscope. All photographs were captured from the holotype (OSTR #8123).

### ﻿Molecular analyses

Total genomic DNA from a single individual was extracted using the Qiagen Mini Spin Plant Kit (Qiagen, Germany). Details concerning DNA quantification and nanopore sequencing are identical to those in the previous studies (Plášek et al. 2023; Sawicki et al. 2023), but due to lower than required for native DNA sequencing amount of extracted DNA, the low input protocol for library preparation was used. The PCR amplification of total DNA was performed using EXP-PCA001 (Oxford Nanoporetech, UK, hereafter ONT) expansion module followed by SQK-LSK114 kit (ONT) protocol. The beads-based post PCR cleaning stage was replaced by column based method using Clean-Up kit (A&A Biotechnology, Poland) and remaining steps and reagents were as recommended in Lingation sequencing V14 - low input by PCR protocol (ONT). Prepared library was sequenced using FLO-MIN114 (ONT) flow cell and sequenced using Minion Mk1C device. Raw reads were basecalled using Dorado 0.5.1 (ONT) using SUP model with enabled duplex read calling. For downstream analyses, reads containing duplex flags were extracted using Samtools software (Danecek et al. 2021). Application duplex high quality reads (Q>30) allow assembling error-free plastomes using exclusively nanopore sequencing technology (Sawicki et al. 2024).

Obtained raw reads were trimmed using porechop 0.2.4 and assembled using Flye 2.91 assembler (Kolmogorov et al. 2019), which produced complete, circularized plastome contigs. The complete chloroplast genome was annotated using previously published *Orthotrichum* Hedw. sequences (Mizia et al. 2019; Frangedakis et al. 2021; Plášek et al. 2023) as references in Geneious Prime 2023.2.1 software (Biomatters, Auckland, New Zealand) and deposited in GenBank under PP274123 accession number. The newly sequenced genome was aligned with previously analyzed dataset of Orthotrichaceae plastomes (Plášek et al. 2023) using MAFFT 7.52 (Katoh and Standley 2013). The second copy of IR was removed from subsequent analyses and ambiguously aligned regions were trimmed by Gblocks 0.91 (Talavera and Castresana 2007). The plastome map was drawn using Chloe web server (https://chloe.plastid.org/).

Chloroplast sequences of 22 specimens of Orthotrichaceae, including seven from *Orthotrichum* were used for phylogenetic analysis. Phylogenetic analysis was carried out using the Bayesian inference (BI) according to model and parameters used in previous study (Plášek et al. 2023). The discrete molecular diagnostic characters (MDCs) for each species of *Orthotrichum*, were calculated according to the Jörger and Schrödl (2013) approach using FASTACHAR 0.2.4 (Merckelbach and Borges 2020).

## ﻿Results

### ﻿Taxonomic treatment

#### ﻿ Orthotrichum camanchacanum

Taxon classificationPlantaeOrthotrichalesOrthotrichaceae

Plášek, Sawicki & Osorio sp. nov.

2E6E3165-D7E8-5322-8988-A04F04ED3CEC

##### Diagnosis.

*Plantae olivacea, obscure viridis, usque ad 1-cm altae. Folia erecta, lineari-lanceolata, carinata, apicibus longis acuminatis. Capsulae emergentes, cylindricae. Stomata cryptopora. Vaginula dense pilosa cum capilli longi. Peristomium duplex, exostoma 8 paribus dentium siccitate erectum, endostoma 16 segmentis. Calyptra dense pilosa. Sporae 19–24 µm, leniter papillosae*.

##### Type.

Chile, Región de Coquimbo (Region IV), Provincia del Elqui, Comuna de Coquimbo, 2 km southeast of Totoralillo town, GPS: 30°04'26"S, 71°21'13"W (-30.073972, -71.353583), on hills profoundly influenced by the humidity generated by camanchaca, vegetation formed mainly by shrubs (*Adesmiaargyrophylla* Phil. and Echinopsischiloensissubsp.chiloensis (Colla) H.Friedrich & G.D.Rowley), moss was found epiphytically on bark of shrubs, leg. F. Osorio *4378*, 10 Aug 2021, holotype (OSTR #8123); isotype (VALD *s.n.*).

##### Description.

Plants in dense tufts to 1 cm tall, olive green above, dark green to brown below (Fig. 2). Stem moderately branched, branches up to 5 mm long. Rhizoids well developed, mainly at base of stems. Stem leaves erect to slightly recurved when dry, spreading to slightly recurved when moist; in upper third linear-lanceolate, long acuminate, 3.0–4.1 × 0.3–0.5 mm, carinate; costa ending just below apex or more often excurrent in hyaline long arista. Leaves in the middle and lower part of the stem wider and shorter, ovate-lanceolate, acuminate, 3.0–3.6 × 0.4–0.7 mm, carinate; costa ending just below apex or rarely excurrent in short aristae. Lamina of stem leaves unistratose, margins entire, recurved from the base to two-thirds of the leaf. Hyaline aristae rectangular, formed by (1–)2–3 gradually elongating cells, from 50–65 to 90–115 × 8–10 µm. Upper laminal cells isodiametric to short elongate, (9–)10–15 × 8–11 µm, fairly thick-walled, with one low papillae on both side; basal laminal cells elongate rectangular to rhomboidal, thick-walled, (18–)20–45 × 10–12 µm, smooth. Alar cells slightly differentiated. Sexual condition goniautoicous. Perichaetial leaves differentiated, ovate-lanceolate, acuminate, significantly shorter than stem leaves, only 1.7–2.2 × 0.5–0.8 mm; upper cells forming conspicuous hyaline (sometimes denticulate) apex (Fig. 3). Seta 1.1–1.5 mm long, ochrea up to 1/5 of the seta, vaginula densely hairy with 0.8–1.9 mm long, single-rowed or sometimes double-rowed, smooth hairs, which usually reach the base of the urn (Fig. 4). Capsule emergent; cylindric to oblong-ovoid, about 1.8–2.2 mm long, yellowish brown, slightly constricted below the mouth when dry. Exothecial cells differentiated mainly in the upper half of capsule, urn strongly furrowed when dry. Stomata cryptopore, scattered in the lower part of the urn, more than half covered by subsidiary cells. Peristome double (Figs 4, 5), preperistome absent. Exostome of 8 pairs of teeth, yellow to light brown, erect-spreading when dry. The outer peristome layer (OPL) ornamentation formed by dense papillae below and a mixture of papillae and distinct striae above. The primary peristome layer (PPL) finely and evenly ornamented by vermiculous lines, slightly also with low papillae. Endostome segments 16, almost as tall as exostome, reflexed when dry; 8 main segments double-rowed and 8 intermediate thinner, single-rowed, somewhat shorter or completely broken in matured capsules. The inner peristome layer (IPL) smooth or ornamented indistinctly by vermiculous lines in the lower part. Calyptra conic-oblong, more or less plicate, yellowish with longitudinal brown stripes, apex red-brown, densely hairy with long, yellowish, smooth hairs. Lid conic, apiculate. Spores light brown, 19–24 µm, densely papillose. Asexual reproduction not observed.

##### Distribution and ecology.

**Figure 1.**
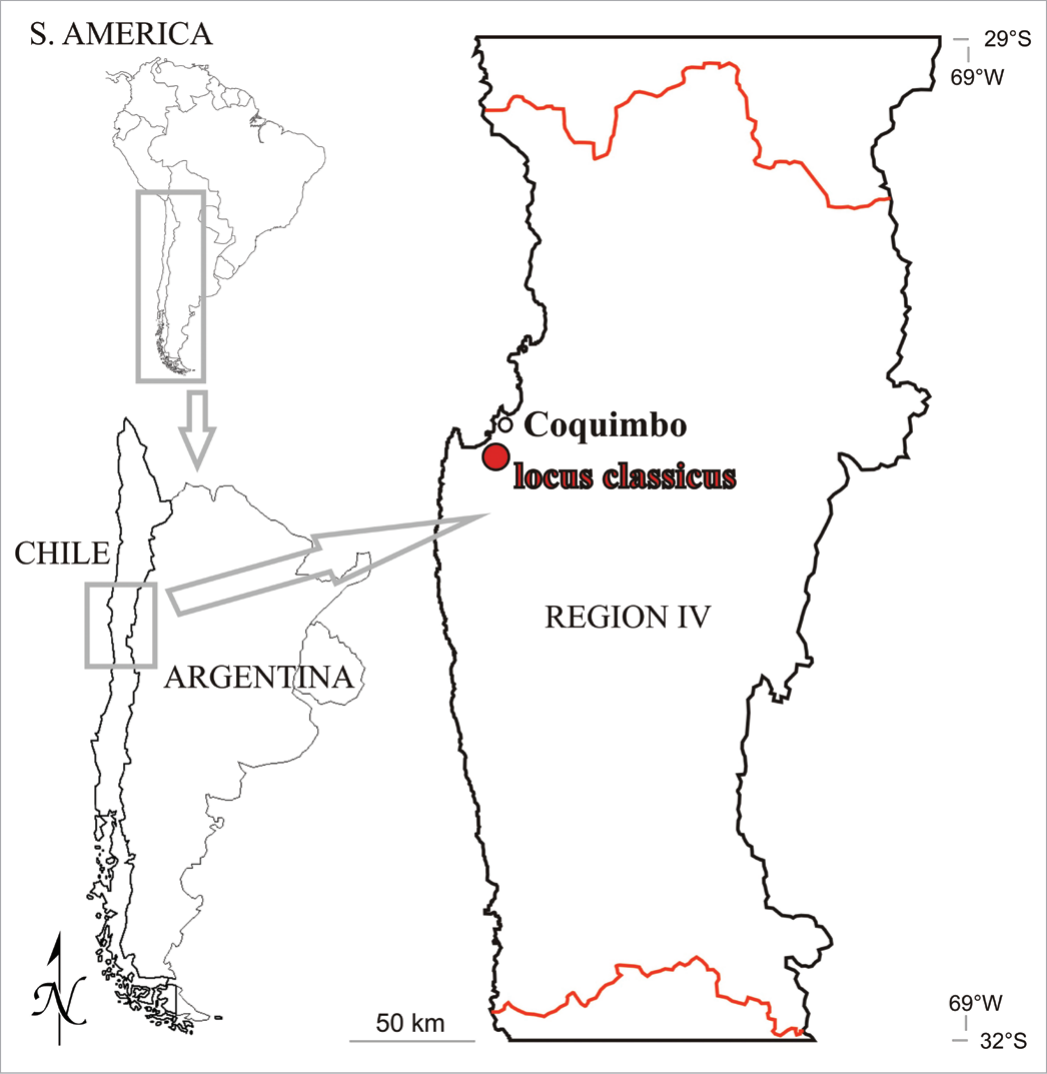
The map illustrates the geographical location where *Orthotrichumcamanchacanum* was discovered.

Moss *Orthotrichumcamanchacanum* was discovered growing epiphytically on the bark of shrubs within vegetation predominantly composed of shrubs such as *Adesmiaargyrophylla* and Echinopsischiloensissubsp.chiloensis. This epiphytic growth pattern suggests a specific ecological niche for this moss within the ecosystem. See map (Fig. 1) for a visual representation of the study area. The territory under investigation lies within the Coquimbo region, characterized by numerous transverse valleys. Notably, the Elqui valley, where *Orthotrichumcamanchacanum* was observed, is situated within this region. The environmental conditions in the study area are influenced by the Andes mountain range, contributing to a steppe-like climate. This climate is typified by sparse vegetation, consisting primarily of shrubs and scrubby vegetation. Precipitation, the heaviest of which occurs during the winter months, further shapes the ecological dynamics of the region.

**Figure 2.**
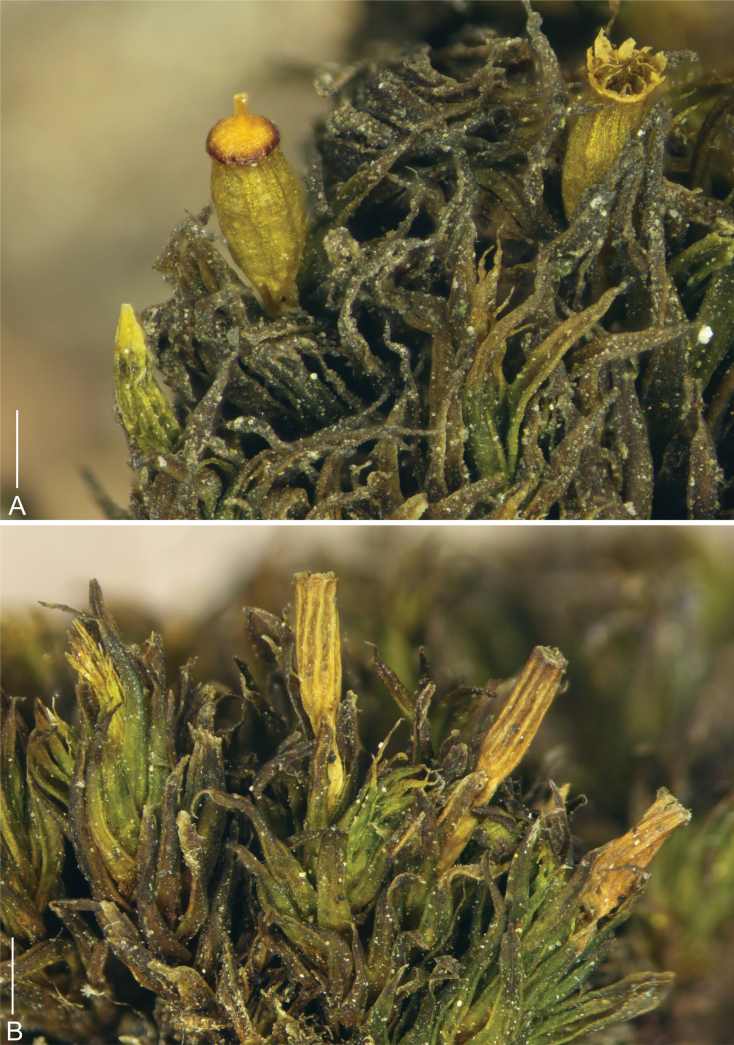
Macro photographs of *Orthotrichumcamanchacanum*. View on fertile plants: **A** plants with capsules at various stages of development: young immature capsule (left), capsule closed by lid (middle), and mature capsule with open peristome (right) **B** dried furrowed capsules emergent from long leaves. Scale bars: 1 mm (**A, B**). Photographs were taken from the holotype (OSTR #8123).

**Figure 3.**
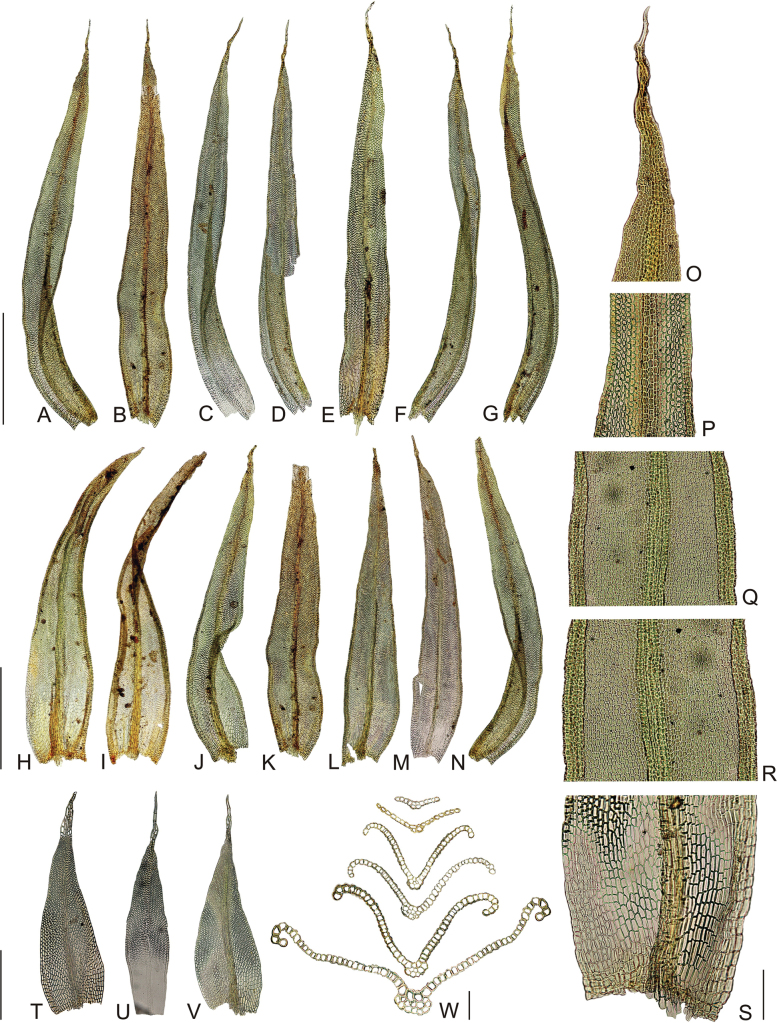
Micro photographs of *Orthotrichumcamanchacanum* leaves **A–G** leaves from upper part of a stem (linear-lanceolate with remarkable narrow hyaline apex) **H–N** leaves from middle and lower part of a stem (many of them are damaged and without apical part) **O–S** detail views of laminar cells, abaxial views (**O** apical part **P** upper part **Q** middle part **R** lower part and **S** base of leaf) **T–V** perichaetial leaves **W** leaf sections (from apical to basal part). Scale bars: 1 mm (**A–N**); 100 µm (**O–S**); 0.5 mm (**T–V**); 50 µm (**W**). Photographs were taken from the holotype (OSTR #8123).

**Figure 4.**
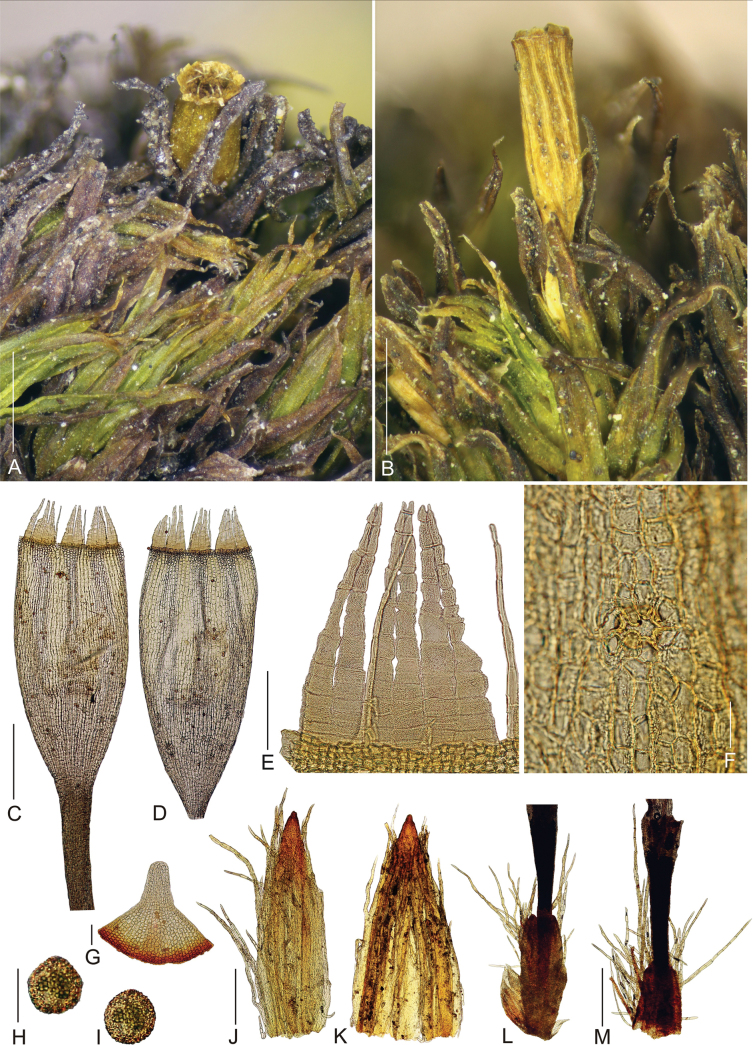
Macro and micro photographs of *Orthotrichumcamanchacanum* sporophyte characters **A, B** view on mature capsules **C, D** mature capsule with peristome **E** detail of peristome **F** stoma (immersed) on capsule urn **G** lid **H, I** spores **J, K** calyptra covered by long hairs **L, M** hairy vaginula. Scale bars: 1 mm (**A, B**); 0.5 mm (**C, D**); 100 µm (**E**); 50 µm (**F**); 0.1 mm (**G**); 20 µm (**H, I**); 0.5 mm (**J–M**). Photographs were taken from the holotype (OSTR #8123).

**Figure 5.**
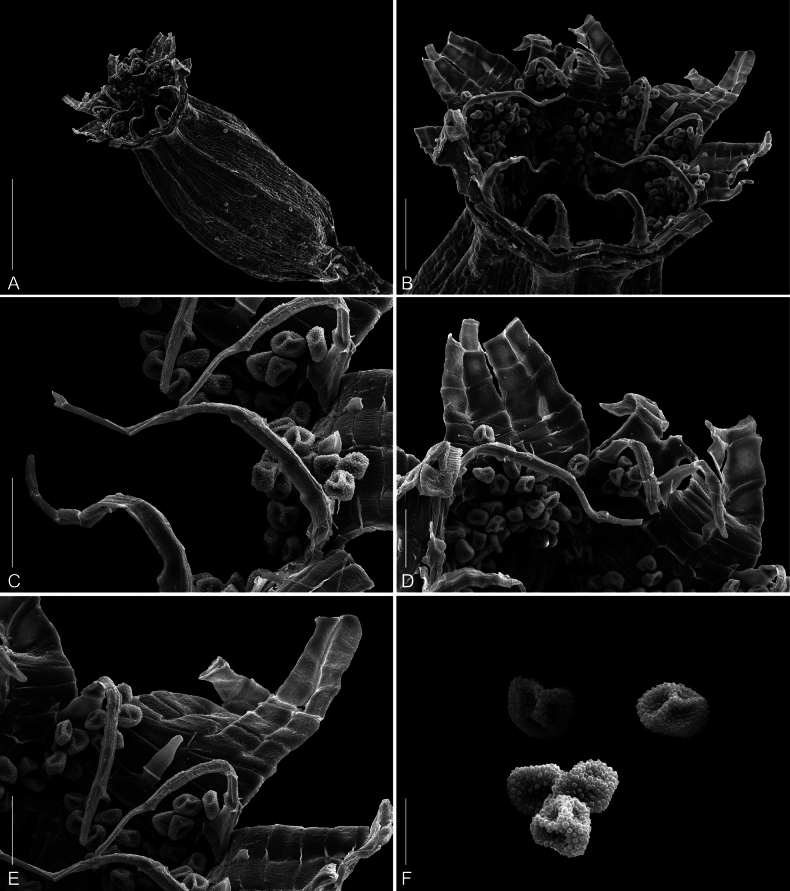
SEM photographs of *Orthotrichumcamanchacanum***A** capsule **B** double peristome **C–E** detailed view on exostome teeth and endostome segments **F** spores. Scale bars: 500 µm (**A**); 100 µm (**B**); 50 µm (**C–E**); 20 µm (**F**). Photographs were taken from the holotype (OSTR #8123).

##### Etymology.

The authors are delighted to name the species (camanchacanum) after “camanchaca”, a term derived from Aymara native language, signifying marine cloud (fog). Forming along the Chilean coast as a cloud, the camanchaca transforms into a dense fog as it moves inland towards the mountains. This fog provides the humidity essential for plant survival.

### ﻿Molecular survey

**Figure 6.**
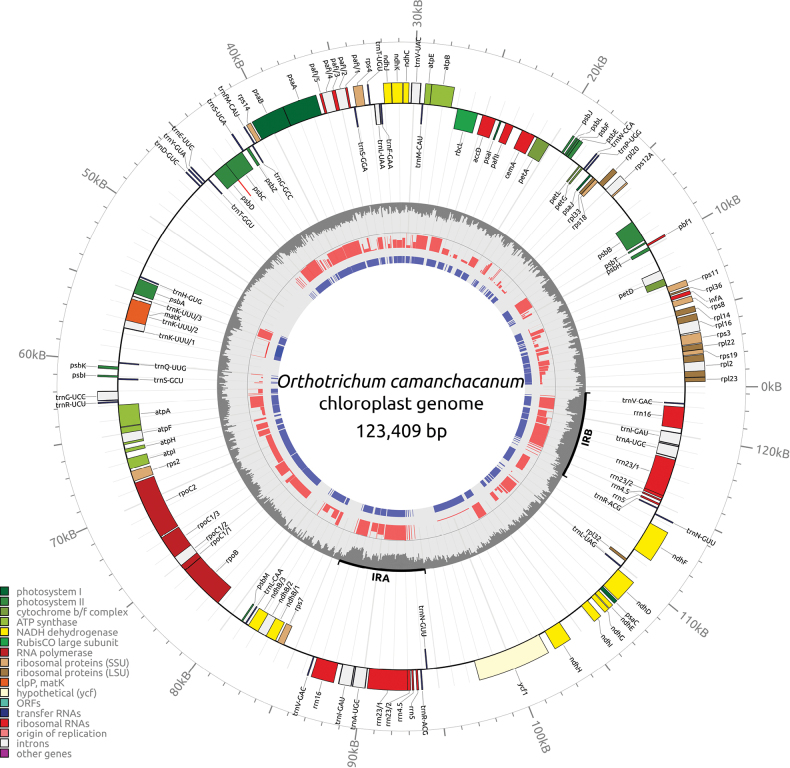
Chloroplast genome of newly described *Orthotrichumcamanchacanum*. Gray inner circle indicates GC content. Red bars indicate normalized score form reference genome (*Orthotrichumrogeri*) while blue bars incidence percentage of reference genome features.

Chloroplast genome of *Orthotrichumcamanchacanum* sp. nov. was 123,409 bp long and had a typical quadripartite structure with one small single-copy (SSC), one large single-copy (LSC), and two inverted repeats (IR). The use of third-generation sequencing did not identify any structural heteroplasmy associated with inversions in the SSC region. The newly sequenced plastome comprises 82 gene encoding proteins (including hypothetical chloroplast reading frames like ycf1, 2, 3, 4, 12, and 66), 32 transfer RNA (tRNA), and four ribosomal RNA (rRNA) genes. Notably, the rps12 gene is split into two separate transcription units, namely 5’-rps12 and 3’-rps12, and their transcripts undergo trans-splicing (Fig. 6).

Analysis of molecular diagnostics characters (MDCs) revealed 641 SNPs characteristic for *Orthotrichumcamanchacanum* followed by 646 for *O.rogeri* and 890 in the case of *O.cupulatum*. Higher numbers of MDCs were detected for *O.crenulatum* and *O.stellatum*, 932 and 1304 respectively.

**Figure 7.**
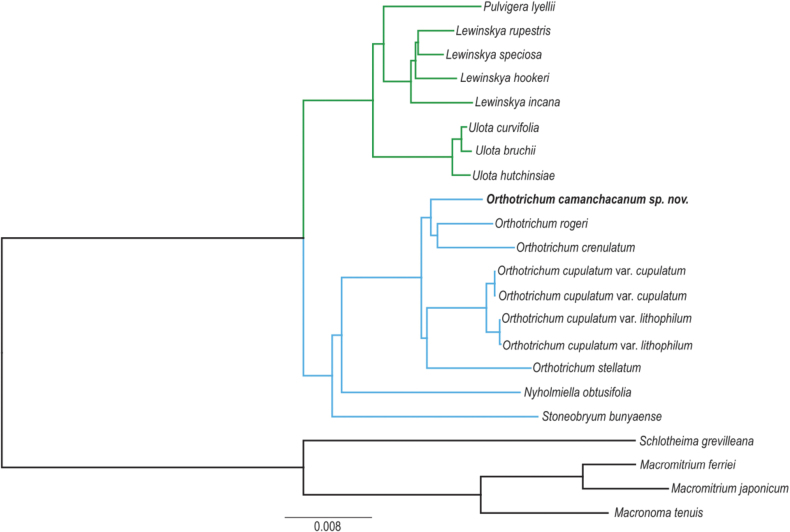
The Bayesian inference tree based on complete chloroplast genomes. All clades have maximum PP values (1.0).

The phylogenetic relationships based on complete plastomes sequences results in a tree (Fig. 7) with all nodes maximally supported by Bayesian posterior probabilities (1.0).The Orthotrichaceae species were found to split into two separate groups, or clades. The first clade includes genera from the Lewinskyinae subgroup, namely *Lewinskya*, *Pulvigera*, and *Ulota*. The second clade consists of genera from the Orthotrichinae subgroup, which are *Nyholmiella*, *Stoneobryum* D.H. Norris & H. Rob., and *Orthotrichum* s. str. Upon analysis, each of these genera was confirmed to be monophyletic. Within these groupings, *Ulota* was identified as the closest relative to the combined *Pulvigera*/*Lewinskya* clade. Similarly, *Nyholmiella* was found to be most closely related to *Orthotrichum*. The newly described *Orthotrichumcamanchacanum* resolved as sister to *O.crenulatum*/*O.rogeri* clade.

## ﻿Discussion

The combination of characters of the newly described species, *Orthotrichumcamanchacanum*, is distinctive, making it easily recognizable. Upon initial observation, the most notable feature of the gametophyte is the shape of the stem leaves, particularly those in the upper third of the stem. They exhibit a linear-lanceolate form with a narrow apex that terminates in a long, hyaline arista (Fig. 3A–G, O). When comparing the shape of the stem leaves, the majority of the Chilean species within the *Orthotrichum* genus generally have ovate-lanceolate leaves (Table 2) with shorter apices. Only *O.inclinatum* (in Lewinsky 1984 as *O.compactum* Dusén) shares a similarly linear-lanceolate leaf shape, but it differs from *O.camanchacanum* in other gametophyte characters, such as the absence of a long aristate hyaline apex in the stem leaves, a naked vaginula, and the absence of differentiated perichaetial leaves. Adding sporophyte characters, then, *O.inclinatum* has long exserted capsule, naked vaginula and different combination of peristome (8+8).

**Table 2.** Comparison of diagnostic characters in Chilean species of the genus *Orthotrichum*. Data for the newly described species are presented in bold.

Species / Diagnostic characters	leaf shape	leaf margin	lamina	perichaetial leaves	vaginula	capsule position	exostome teeth	endostome segments	preperistome	spore size (µm)	calyptra	asexual repr.
*Orthotrichumanomalum*	lanceolate	revolute	unistratose	not differentiated	occasionally hairy	long exserted	8 pairs	absent	present	14–18	hairy	not observed
*O.assimile*	ovate-lanceolate	revolute	unistratose	not differentiated	naked	emergent	8 pairs	8 (or 16)	absent	15–20	naked or sparsely hairy	gemmae (uncommon)
***O.camanchacanum***	**linear-lanceolate**	**recurved**	**unistratose**	**differentiated**	**densely hairy**	**emergent**	**8 pairs**	**16 segments**	**absent**	**19–24**	**densely hairy**	**not observed**
O.cupulatumvar.austroamericanum	ovate-lanceolate	recurved	unistratose	not differentiated	naked	shortly emergent	16 teeth	mostly absent	present	15.5–20	hairy	not observed
O.diaphanumvar.podocarpi	ovate-lanceolate	revolute	unistratose	not differentiated	naked	immersed to emergent	8 pairs (splitting)	16 segments	absent	18–22	hairy	gemmae
*O.freyanum*	ovate-lanceolate	revolute	unistratose	not differentiated	naked	emergent to shortly exserted	8 pairs	8 segments	absent	12–15	hairy	gemmae
*O.gigantosporum*	ovate-lanceolate	plane or slightly recurved	unistratose	differentiated	hairly	long exserted	16 teeth	16 segments	absent	31–34 (–40)	naked	not observed
*O.inclinatum*	linear-lanceolate	plane or revolute	unistratose	not differentiated	naked	long exserted	8 pairs	8 segments	absent	13–19	with few scattered hairs	gemmae (occasionally)
*O.perexiguum*	narrow lanceolate	plane	bistratose	differentiated	naked	emergent to just exserted	8 pairs	8 segments	absent	14–16	naked	not observed
*O.tristriatum*	ovate-lanceolate	broadly revolute	unistratose	differentiated	naked	emergent to shortly exserted	8 pairs	8 segments	absent	14–18	with few scattered hairs	not observed
*O.truncatum*	ovate-lanceolate	reflexed	unistratose	not differentiated	hairly	shortly exserted	8 pairs	8 segments	mostly absent	20–23	with few scattered hairs	not observed

Although *Orthotrichumperexiguum* also exhibits a narrow and lanceolate leaf shape (Lewinsky 1984), this species is notably small, reaching only up to 0.5 cm. Moreover, it can be distinguished by a bistratose laminal in its marginal parts, a naked vaginula, and a different peristome combination (8+8).

The perichaetial leaves produced by *Orthotrichumcamanchacanum* are distinctive and markedly different when compared with all South American species of this genus. Notably, they possess a unique apex formed by a group of conspicuous hyaline cells (Fig. 3T–V). In contrast, the perichaetial leaves of other Chilean *Orthotrichum* species that produce them (such as *O.gigantosporum*, *O.perexiguum*, and *O.tristriatum*), are similar in shape to the stem leaves, differing mostly in their smaller size (Lewinsky 1984).

The hairiness of the vaginula is considered a distinctive taxonomic character for identifying *Orthotrichum* species (Lewinsky 1993). However, in some European species, this character has not always proven suitable due to its considerable variability (Plášek and Sawicki 2010). Among the Chilean species, three exhibit a distinctly hairy vaginula (*O.camanchacanum*, *O.gigantosporum*, and *O.truncatum*), while in one other species (*O.anomalum*), its hairiness was only occasionally noted (Lewinsky 1984; Lewinsky and Deguchi 1989). In *O.camanchacanum*, the hairs in vaginula are dense and long, often reaching the base of the urn or clearly visible among upper stem leaves.

When comparing the sporophyte characters of the Chilean species within the genus *Orthotrichum*, most of them, including the newly described species, have emergent or shortly exserted capsules. However, three species (*O.anomalum*, *O.gigantosporum*, and *O.inclinatum*) produce significantly exserted capsules on a long seta (Lewinsky 1984; Lewinsky and Deguchi 1989). In *Orthotrichum* species, the peristome is of the arthrodontous type and primally diplolepidous. Typically, it consists of eight pairs of exostome teeth, occasionally splitting into 16, and eight or sixteen endostome segments. The combination of the number of teeth and segments, along with their surface ornamentation, is a crucial taxonomic feature for both the genus and the entire family Orthotrichaceae (Lewinsky 1993). In the case of *O.camanchacanum*, its peristome is formed by eight pairs of exostome teeth with the outer peristome layer densely papillose below and a mixture of papillae and striae in the upper part. A similar OPL surface is observed in *O.alpestre* Bruch & Schimp., a European species not found in South America (cf. Lara et al. 2009; Plášek and Ochyra 2020). Regarding the endostome of the newly described species, it consists of 16 thin segments, which are delicate and often partially broken. However, in well-developed capsules, all the segments are significantly long, almost reaching the length of the exostome teeth. Only two other Chilean species, *O.assimile* and *O.tristriatum*, have similarly long and thin endostome segments, but both of them generally have a total of eight segments.

It is widely acknowledged that species within the genus *Orthotrichum* have immersed stomata, distinguishing them from representatives of the related genus *Lewinskya*, whose urns possess superficial stomata (Lewinsky 1993). Despite this, some species across both genera exhibit macroscopic similarities. Paradoxically, in the case of *Orthotrichumcamanchacanum*, certain South American *Lewinskya* species share a closer resemblance than *Orthotrichum* species, especially those with distinctly long and narrow leaves culminating in a markedly narrow apex, e.g. *L.elongata* (Taylor) F. Lara, Garilleti & Goffinet, *L.johnstonii* (E.B. Bartram) F. Lara, Garilleti & Goffinet or *L.mandonii* (Schimp. ex Hampe) F. Lara, Garilleti & Goffinet (cf. Lewinsky 1984). However, aside from the already mentioned distinct positions of the stomata, these *Lewinskya* species also differ in a clear combination of peristome parts. This marked difference in characters prevents any confusion between the newly described species and representatives of *Lewinskya* species.

The territory where the new species was collected possesses distinctive geographical and climatic conditions. The entire Coquimbo region features several transverse valleys that cut across perpendicular to the main Andes mountain chain, carving through the country horizontally (Alaniz and Carvajal 2019). Among these valleys is situated the Elqui interion region, where *Orthotrichumcamanchacanum* was collected. The Andes contribute the Región de Coquimbo, providing a steppe-like climate characterized by bushy, scrubby vegetation and heaviest precipitation in the winter (Moreira-Muñoz 2011). On the coast, especially on the coastal mountain range influenced by the moderating effect of the ocean, an abundance of clouds and coastal fog, known as camanchaca, fosters the growth of Chile’s northernmost forests (Moreira-Muñoz 2011). Inland, the climate becomes warm and typically dry (Alaniz and Carvajal 2019). This varied landscape relief combined with a unique climate may have significantly contributed to the speciation of this new species. The moss *O.camanchacanum* exhibits morphological characters that distinguish it from related species. The fact that it has not been found despite intensive research carried out in Chile since 2011 by the first author may be attributed to its geographical isolation and consequently a very limited area of occurrence. It seems to be endemic to this region of Chile. This situation is not unique; similar examples exist, such as the moss *Lewinskyajohnstonii*, which also possesses unique morphological features and is limited to the National Park Fray Jorge and its close surroundings (Lewinsky 1984). This area similarly exhibits a specific and characteristic climate.

## Supplementary Material

XML Treatment for Orthotrichum camanchacanum

## ﻿Additional information

### Author ORCIDs

Vítězslav Plášek https://orcid.org/0000-0002-4664-2135

Jakub Sawicki https://orcid.org/0000-0002-4759-8113

Felipe Osorio https://orcid.org/0009-0001-0759-920X

Monika Szczecińska https://orcid.org/0000-0002-5377-4304

Hana Režnarová https://orcid.org/0009-0004-2168-4015

### Data availability

All of the data that support the findings of this study are available in the main text.
